# Stress and Perception of Emotional Stimuli: Long-term Stress Rewiring the Brain

**DOI:** 10.29252/NIRP.BCN.9.2.107

**Published:** 2018

**Authors:** Reza Khosrowabadi

**Affiliations:** 1. Institute for Cognitive and Brain Sciences, Shahid Beheshti University, Tehran, Iran.

**Keywords:** Long-term stress, Effective connectivity, Electroencephalography (EEG), Emotion

## Abstract

**Introduction:**

Long-term stressful situations can drastically influence one’s mental life. However, the effect of mental stress on recognition of emotional stimuli needs to be explored. In this study, recognition of emotional stimuli in a stressful situation was investigated. Four emotional conditions, including positive and negative states in both low and high levels of arousal were analyzed.

**Methods:**

Twenty-six healthy right-handed university students were recruited within or after examination period. Participants’ stress conditions were measured using the Perceived Stress Scale-14 (PSS-14). All participants were exposed to some audio-visual emotional stimuli while their brains responses’ were measured using the Electroencephalography (EEG) technique. During the experiment, the subject’s perception of emotional stimuli is evaluated using the Self-Assessment Manikin (SAM) questionnaire. After recording, EEG signatures of emotional states were estimated from connectivity patterns among 8 brain regions. Connectivity patterns were calculated using Phase Slope Index (PSI), Directed Transfer Function (DTF), and Generalized Partial Direct Coherence (GPDC) methods. The EEG-based connectivity features were then labeled with SAM responses. Subsequently, the labeled features were categorized using two different classifiers. Classification accuracy of the system was validated by leave-one-out method.

**Results:**

As expected, performance of the system is significantly improved by grouping the subjects to stressed and stress-free groups. EEG-based connectivity pattern was influenced by mental stress level.

**Conclusion:**

Changes in connectivity patterns related to long-term mental stress have overlapped with changes caused by emotional stimuli. Interestingly, these changes are detectable from EEG data in eyes-closed condition.

## 1. Introduction

Long-term mental stress, one of the most important issues in today’s society, causes a variety of health problems. Our cognition, perception, and decision making can also be affected by long-term stress. This paper investigates that how stress can influence our perception to external emotional stimuli.

There is an association between mood (e.g. Long-term stress), emotional states and homeostatic changes (Craig, 2003). When a stimulus is perceived, the brain initiates a reverse course of actions that releases different biochemical compounds throughout the body to bring the body back into a balance state. A balance state is a metabolic equilibrium between stimulating and tranquilizing chemical forces in the body. If either stimulating or tranquilizing chemical forces dominates the other without relief, then an on-going state of internal imbalance is experienced. This condition is known as stress which can have a serious consequence on the brain cells. Stress is defined as an on-going and unrelieved imbalance between stimulating and tranquilizing biochemicals. Thus, this condition may damage brain cells. Stress can be acute (short-term) or long-term. Acute stress is usually not a health risk. In contrast, long-term stress causes a wide variety of health problems (Lundberg, 2008). As long-term stress lasts longer than emotional states (Ekman, 1999), the resulting homeostatic instability can be considered as a base for emotional states. This hemostatic instability can potentially affect subject’s emotional perception.

In this study, long-term mental stress level of a subject is estimated by PSS-14 questionnaire (Cohen, Kamarck, & Mermelstein, 1983) that comprises 14 items. In each item, subject is asked how often he/she has experienced certain occurrence of a stressful situations during the last month. Response for each item is scored from 0 to 4, thus total response scores range from 0 to 56. A higher response score correlates with a higher level of mental stress. However, there is no score cut-off point and comparisons are only sample-wise. In this study, subjects’ responses to PSS-14 had mean and standard deviation of μ=24, σ=6.7 respectively. Subjects with a score lower than μ-σ/2=21 were considered to be relatively stress-free whereas those with a score higher than μ+σ/2=28 were considered to have long-term stress.

Furthermore, emotions are psychophysiological phenomena associated with a wide variety of subjective feelings and observable behaviors. In general, perception of an emotional stimulus comprises cognitive process, subjective feeling, and physiological reactions. Subsequently, an expression in response to the stimulus is set by a series of chemical releases and reactions (Baumgartner, Esslen, & Jäncke, 2006). Physiological changes due to either perception or expression are detectable (Khosrowabadi, Hiok Chai, Wahab, & Kai Keng, 2010; Olofsson, Nordin, Sequeira, & Polich, 2008) and studies have shown that EEG also can be used to reveal them (Farquharson, 1942). In order to find EEG signatures related to perception of an emotional stimulus, subject’s feeling of the presented stimulus should be identified. Therefore, a way has to be described for distinction of subjects’ emotional states.

Theories in emotion have suggested a number of basic emotions. Basic emotions are defined as emotions that are common across cultures and selected by nature because of their high survival factors. Commonly accepted basic emotions are: happy, sad, fear, anger, surprise and disgust. Accordingly, complex emotions are then formed by combination of some basic emotions Theories in emotion have suggested several mutual emotions across cultures, aka: basic emotions, and some complex emotions that consist of elements of basic shared emotions, such as happiness, fear, anger, surprise, and disgust. These emotions were preserved by natural selection as they were necessary tools for survival (Ekman, 1999; Ortony & Turner, 1990). However, there is still no coherent definition for basic emotions. Therefore, to overcome this issue, it has been suggested to categorize emotions based on their valence and arousal levels (Ekman, 1999; Russell, 1980).

In this dynamic representation method, valence varies from unpleasant to pleasant and arousal varies from low (calm) to high level of excitement. In addition, basic emotions can also be differentiated in the valence–arousal plan. Consequently, four types of emotional stimuli including positive and negative either with low or high level of excitement were investigate in this study. However, it should be noted that different subjects may experience different emotions while exposed to the same stimulus. Therefore, Self-Assessment Manikin (SAM) questionnaire (Lang, 1980) was used to find out about a subject’s true feelings. In the next step, the SAM responses were used to categorize EEG features.

In this study, differentiable connectivity patterns of emotional states were investigated. The connectivity patterns between eight brain regions were estimated based on EEG data. It has been shown in our previous studies that connectivity-based features are superior to other existing feature extraction methods for recognition of emotions by EEG signals. The extracted features were then labeled by subjects’ SAM responses. Afterwards, the EEG features correlates on emotional states were classified in a supervised manner. A significant change in the accuracy of recognizer system was observed after categorizing the subjects to stressed and stress-free groups. Hence, it was hypothesized that long-term stress disrupts patterns of connectivity between the brain regions. To investigate the matter, possibility of recognition of long-term stress level from EEG data was explored. The subjects participated in our study were categorized to stressed and stress-free groups based on their EEG data acquired in resting state (eyes-closed condition). The result of resting state also confirmed that the long-term stress distracts patterns of connectivity among different brain regions. Interestingly, this effect on neural activities was detectable in EEG signals acquired in eyes-closed condition.

The remainder of this paper is structured as follows. Section II describes experimental protocol. Section III describes the signal processing of EEG, feature extraction and classification part. Section IV presents the experimental results. Section V concludes the paper. The reminder of this report is structured in four sections. Section two explains the launched experimental protocol. Section three discusses the method of EEG signal processing, feature extraction and classification. Section four is devoted to the experimental results. Section five concludes the research.

### 1.1. Experimental design

The following section describes the experimental design to collect EEG correlates on emotions.

### 1.2. Emotion elicitation

Studies have shown that elicitor (subject elicited vs. event elicited), setting (controlled condition in the lab vs. real world), focus (expression vs. perception), and subject awareness (open recording vs. hidden recording) are factors that can influence the emotion elicitation results (Picard, Vyzas, & Healey, 2001). Subject elicited category refers to the instruction given to the subject to express a specific emotion (for example to mimic the facial expression of happiness), or recalling past emotional episodes. Event-elicited category refers to use of some images, sounds, video clips or any emotionally evocative stimuli. The International Affective Picture System (IAPS) (Lang, Bradley, & Cuthbert, 2005), International Affective Digitized sound System (IADS) (Bradley, Lang, University of Florida, & National Institute of Mental Health, 1999) Bernard Bouchard’s synthesized musical clips (Vieillard et al., 2008), and Gross and Levenson’s movie clips (Gross & Levenson, 1995) are standard data sets used for elicitation of emotions. Although touching, smelling, and tasting are also known to influence human emotions, these are less studied in the literature (Kulish, Sourin, & Sourina, 2007).

**Figure 1.**
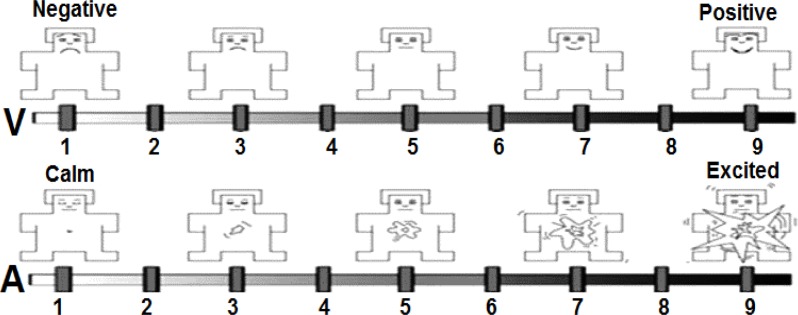
2D Self-Assessment Manikin questionnaire

Therefore in this study, a combination of arousing pictures from IAPS and synthesized musical excerpts of Bernard Bouchard were used to elicit emotions. The emotional stimuli data set was evaluated by experts accompanied by average judgments of several people. However, actual emotional feeling of a stimulus may differ from one subject to another based on their experiences. Therefore, even though predefined evaluation labels were available, self-assessments questionnaire were also used. The subjects were asked to rate their feelings about the presented stimulus on a SAM questionnaire (Figure 1). After selection of emotional stimuli, a strategy for sequence and durations of emotional stimuli should be selected.

### 1.3. Experimental protocol

**Figure 2.**
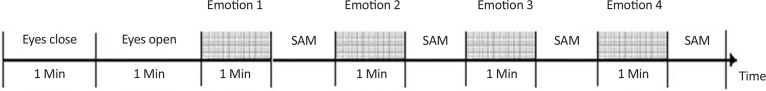
Paradigm for inter-subjects experiment

The durations of affective stimuli are mainly defined based on study categories. Three major categories can be listed as: 1) full blown emotions that last from several seconds to minutes, 2) moods that last from several minutes to hours, and 3) emotional disorders that last from several years to entire lifetime (Cowie & Cornelius, 2003). Hence, this study focuses on full blown emotions (Cowie & Cornelius, 2003) and emotional stimuli were presented for one minute in a counterbalanced and random order. In fact, an emotion recognition system should be ideally able to discriminate the emotional states by EEG as fast as possible (Coan & Allen, 2004). Therefore, a window length of 2 s was used to process the acquired EEG data. Figure 2 shows the protocol of stimuli presentation.

The EEG data were collected while subject was seated in a comfortable chair in a lit and controlled temperature room. Prior to the experiment, the experimental procedure was explained to the subject in the registration room. The subject was asked to fill in a consent form, PSS-14, and handedness questionnaires (Oldfield, 1971). Then, 8 Ag/AgCl electrodes with a reference electrode (Cz) were attached bilaterally on the subject’s scalp. The 10/20 system of electrode placement was used in this study. Next, the EEG data were collected for a 10-min period that comprised 5 min in eyes-closed condition, 1 min in eyes-open condition, and 1 min for each emotional stimulus. All subjects were exposed to 4 emotional stimuli in different levels of arousal and valence as explained in previous section. The visual stimuli were displayed on a 19 inch monitor positioned 1 m away from the participant’s eyes and the audio stimuli were played with speakers with a constant output power. Four emotional stimuli for each participant were presented randomly while their sequences were counterbalanced to ensure each stimulus category was seen by each subject.

The EEG was recorded using the BIMEC device (Brainmarker BV, The Netherlands) with a sampling rate of 250 Hz. The impedance of recording electrodes was monitored for each subject prior to data collection and it was kept below 10 kΩ. The EEG was recorded by the BIMEC device (Brainmarker BV, The Netherlands). It was setup to sample with a rate of 250 Hz. The impedance level aimed to be below 10 kΩ so that the recording electrodes were carefully monitored for each subject prior to data collection and be kept within the impedance limits.

### 1.4. EEG correlates on emotion

As explained before, the valence-arousal plane was used for labeling the EEG data in this study. The subjects’ responses to SAM questionnaires were used to label EEG features.

### 1.5. Subjects

Statistically, a large sample size increases the precision of estimation. Therefore, EEG data were collected from as many as 26 healthy university students (all right-handed, 18–30 years old, 20 male). The preparation for exams during the examination period was considered as a stressful situation. The EEG data were acquired from 15 subjects during the examination period and 11 subjects 2 weeks after their last exam. However, the PSS-14 answers were used to categorize subjects into stressed and stress-free groups. It has been shown that long-term stress can impair subject’s ability to flexibly shift his or her attention by reducing connectivity to an attention-regulating area of the prefrontal cortex (Arnsten, 2009). Such impairment can potentially influence the cognitive process involved in perception of emotional stimuli. Considering the time lasting of long-term mental stress, this effect should be detectable in resting state as well which is explained in the next section.

## 2. Methods

**Figure 3.**
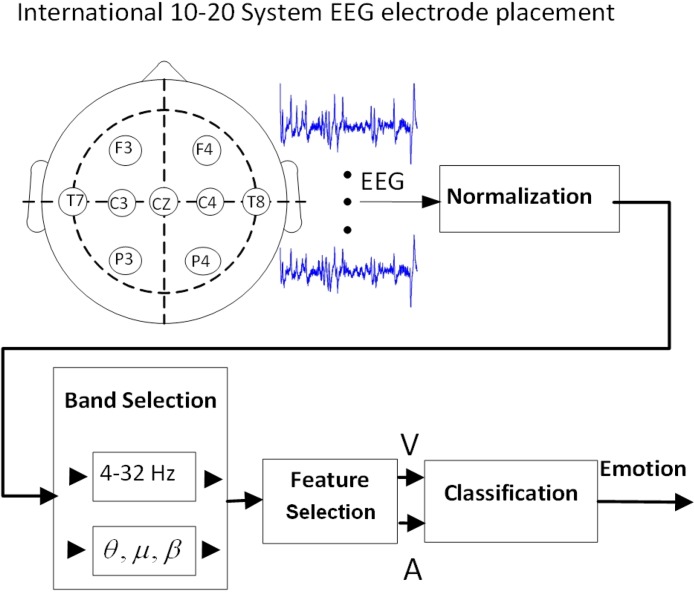
Protocol of data processing

This section describes the methodology used to classify EEG correlates of emotion in this study. The EEG was processed as shown in Figure 3. In the first stage, the EEG data was normalized to amplitude range of. The methodology used to classify EEG correlates of emotion is explained here. Figure 3 shows the EEG processing protocol. Normalization was performed to fit amplitude range of 0 to 1. This scaling helps remove effects of recording conditions for different subjects while it does not change the connectivity features. Since EEG contains a lot of noises and artifacts from bodily movements or eye blinks, noise removal was performed using a band-pass filter in range of 4 to 32 Hz. After, preprocessing, some EEG features were extracted from EEG data.

### 2.1. Feature extraction

The challenging task is to extract distinctive features from EEG that correlates to subject’s emotional states. In the literature, techniques for extracting features from EEG correlates of emotional states include event-related synchronization or desynchronization, event-related potentials, visual-evoked potentials, and quantitative EEG (Aftanas, Varlamov, Pavlov, Makhnev, & Reva, 2001; Coan & Allen, 2004; Frantzidis et al., 2010; Kemp, Gray, Eide, Silberstein, & Nathan, 2002; Olofsson et al., 2008; Petrantonakis & Hadjileontiandis, 2010). Studies have also shown that the cortical-subcortical interactions play an important role in the perception of emotional stimulus (Kober et al., 2008; Lewis & Todd, 2007; Swanson, 2003).

Therefore, brain connectivity features should be very informative. In addition, our previous studies have shown that emotional states can be accurately recognized using these features extracted from EEG data. Therefore, various connectivity features were applied in this study to categorize EEG data collected in different emotional states. Several connectivity measures including phase slope index (Nolte et al., 2008), directed transfer function (Korzeniewska, Manczak, Kaminski, Blinowska, & Kasicki, 2003), and generalized partial directed coherence (Baccald & de Medicina, 2007) were applied in this study.

Mathematical notations used to extract connectivity features are explained here. Inputs, outputs, related variables and functions are presented as follows.

Let *x*_∈_*i*^*n*_*p*_×*n*_*c*_×(*n*_*e*_*n*_*s*_)^ denotes the multi-channel, multi-subject, and single-trial EEG data that are correlated to the valence and arousal ã∈^2×(*n_e_n_s_*)^; whereby nc denotes the number of EEG channels, np denotes the number of time samples per single-trial EEG, ns refers to the number of subjects, and n_e_ is the number of categories of emotional stimuli.

Let, $X={\left[X_{1}^{1},X_{1}^{2},\ldots ,X_{1}^{n_{e}},\ldots ,X_{s}^{t},\ldots ,X_{n_{s}}^{n_{e}}\right]}^{T}$ whereby $X_{s}^{t}\in i^{n_{p}\times n_{c}}$ ×denotes the t^th^ single-trial EEG data from the s^th^ subject also 1≤t≤n_e_ and 1≤s≤n_s_. The numbers nc, ne, ns, np are respectively fixed at 8, 4, 26, 15000 in this study.

Let ${\text{X}}_{\text{S}}^{\text{t}}={\left[{\text{X}}_{\text{S},1}^{\text{t}},{\text{X}}_{\text{S},1}^{\text{t}},\;\;\ldots ,\;\;{\text{X}}_{\text{S},\text{i}}^{\text{t}}\;\;,\ldots ,\;\;{\text{X}}_{\text{S},{\text{n}}_{\text{c}}}^{\text{t}}\right]}^{\text{T}}$, whereby 
X
s,it∈inp×1 denotes a vector that represents the EEG time series from the i^th^ channel and $x_{s,i}^{t}=\left[x_{1},x_{2},\ldots ,x_{n},\ldots ,x_{n_{p}}\right]$ whereby x_n_ denotes the n^th^ sample. Note that a simplified notation of x_n_ was used to denote $x_{s,i}^{t,n}$ in the remainder of this paper.

Let **ã** ∈ *i*^*n_v_* × (*n_e_n_s_)*^ denotes the extracted features that are correlated to **ã** ∈ *i*^2*n* × (*n_e_n_s_)*^, whereby nv denotes the number of features extracted for a particular feature extraction method.

Let $\tilde{a}={\left[v_{1}^{1},v_{1}^{2},\ldots ,v_{1}^{n_{e}},\ldots ,v_{s}^{t},\ldots v_{n_{s}}^{n_{e}}\right]}^{T}$, whereby $v_{s}^{t}\in i^{n_{v}\times 1}$, $v_{s}^{t}=\left[v_{1},v_{2},\ldots v_{k},\ldots v_{n_{v}}\right]$, and *vk* denotes the k^th^ feature.

The basic functions applied to extract the features in this study include rectangular window, autocorrelation, cross correlation, discrete Fourier transform, power spectral density, and cross power spectral density. The mathematical notations of these functions are given as follows:

Let **W∈***i^N^* ^× *n*_c_^, **W** = [**w**_1,_ **w**_2_, …, **w***_i_*, …, **w***_n_c__*]*^T^* whereby **w***_i_* ∈ *i^N^*^×1^, denotes a vector that represents the windowed time series EEG signal from the i^th^ channel.

Let $w_{i}={\text{f}}_{\text{w}}\left(x_{s,i}^{t},s_{t},N\right)$ denotes the rectangular window function of $x_{s,i}^{t}$ given by Equation (1).

(1) $${\text{f}}_{\text{w}}\left(x_{s,i}^{t},s_{t},N\right)=\left[w_{i}^{1},w_{i}^{2},\ldots ,w_{i}^{n},\ldots w_{i}^{N}\right]$$,

where N denotes the window length, s_t_ refers to the start point, and $w_{i}^{n}$ denotes the n^th^ sample of the windowed signal that is the (*s_t_* + *n*)^th^ sample of the i^th^ channel of the EEG data. This study used a sampling frequency Fs=250 Hz, a window length N=512, and a starting point St=500 (2 s).

Let **r***_ii_* = *f_oc_*(**w***_i_*) denotes the autocorrelation function and **r***_ii_* = *f_cc_*(**w***_i_*, **w***_j_*) denotes the cross correlation function between the i^th^ and the j^th^ channel, whereby **r***_ii_* ∈ *i^N^*^×1^ and **r***_ij_* ∈ *i^N^*^×1^. Since w_i_ and w_j_ are stationary with the same length, the autocorrelation and the cross correlation functions for l=0, …, N-1 are respectively given by equations (2) and (3) .

(2) $$f_{oc}(w_{i})=\frac{1}{N-l}\sum_{n=1}^{N-l}w^{*}{}_{i}^{n}w_{i}^{n+1}$$

(3) $$f_{cc}(w_{i},w_{j})=\frac{1}{N-l}\sum_{n=1}^{N-l}w^{*}{}_{i}^{n}w_{j}^{n+1}$$,

where * denotes the complex conjugate.

Let **z***_i_* = *f_DFT_*(**w***_i_*) denotes the Discrete Fourier Transform (DFT), whereby **z***_ii_* ∈ *R^N^*^×1^ transforms the windowed EEG signal w_i_ from time domain to frequency domain.

Let Z^f^_i_ denotes the f^th^ sample of Z_i_ given by Equation (4) .

(4) $${Z^{f}}_{i}=\sum_{N=0}^{N-1}{{𝒲}^{n}}_{i}e^{\frac{-J2\pi fn}{n}}$$,

where $f=[0,1,\ldots ,N-1]\times \left(\frac{F_{s}}{2\times N}\right)$, and J is the imaginary unit.

**s***_ii_* = *N* × *f_DFT_*(**r***_ii_*) denotes the Power Spectral Density (PSD) of w, whereby **s***_ii_* ∈ *i^N^*^×1^ is the DFT of the autocorrelation function r_ii_. **s***_ij_* = *N* × *f_DFT_*(**r***_ij_*) denotes the Cross Power Spectral Density (CPSD) of w_i_ and w_j_, whereby **s***_ij_* ∈ *i^N^*^×1^ is the DFT of the cross correlation function r_ij_. The PSD and the CPSD functions are respectively given by equations (5) and (6) .

(5) $$f_{DFT}\left(r_{ii}\right)=\frac{1}{N}z_{i}{z^{*}}_{i},$$

(6) $$f_{DFT}\left(r_{ij}\right)=\frac{1}{N}z_{i}z_{j}^{*}$$

The Welch’s method, also called the periodogram method, computes the PSD for the entire input signal. This method is performed by dividing the time signal into successive blocks and by averaging the squared-magnitude DFTs of the signal blocks (Welch, 1967).

### 2.2. Directed transfer function

Directed Transfer Function (DTF) is a method that simultaneously takes into account all channels of the process to estimate the activity flow in a given direction as a function of frequency based on the concept of Granger causality (Kaminski & Blinowska, 1991). DTF is closely related to spectral G-causality. The time series EEG signal from channel i (w_i_) is said to Granger cause another channel j (w_j_) if it can be shown by using statistical test (normally F-test) on lagged values of w_i_ (or lagged values of w_j_) and values of w_i_ provide statistically significant information about future values of w_j_. This is fundamental for effective connectivity and here the important point is that this predictability improvement is not reciprocal, i.e. w_i_ may Granger cause w_i_ withoutw_j_ necessarily Granger cause w_i_.

Using linear prediction of future values of multivariate data with an r^th^ order Multivariate Auto Regressive (MVAR) modeling is the simplest way to exploit this idea. Therefore, data can be modeled by Equation (7) .

(7) $$W^{n}=\sum_{r=1}^{m}A^{r}W^{n-r}+{\hat{I}}^{n}$$,

where **w** ∈ ***i****^N^* ^_*c*_× 1^, and

(8) $$W^{n}={\left[W_{1}^{n},\ldots ,W_{i}^{n},\ldots ,W_{n_{c}}^{n}\right]}^{T}.$$

**î** ∈ *^N^* ^_*c*_× 1^ is the matrix of innovation process (a zero-mean Gaussian noise with an estimated covariance matrix σ). Each vector of **w***_i_*, **î***_i_* is considered as an element of w and **î**, respectively. **A***^r^* ∈ *i* ^*n*_c_ × *n_c_*^ is coefficients matrices, each corresponding to a specific lag r and m representing the order of AR model. The diagonal elements of σ (*Ó_ii_*) measure the remaining error when the future values of w_j_ are modeled simultaneously with all the time series values. To analyze the EEG signal, frequency decompositions are often of interest. Therefore, Geweke (Geweke, 1982) proposed using Fourier methods to examine G-causality in the spectral domain which is computed as follows. Equation (9) is computed by transforming Equation (7) to the frequency.

(9) $$\sum_{r=0}^{m}A^{r}w^{n-r}={\hat{I}}^{n}\overset{\mathrm{Fourier}}{\underset{\mathrm{transform}}{\to }}A^{f}W^{f}={\hat{I}}^{f}$$

By recasting Equation (9) into the transfer function format, we obtain **W***^f^* = **H***^f^***ξ***^f^*, where the transfer function (prediction error filter representation) is given by Equation (10) .

(10) $$H^{f}={\left({\bar{A}}^{f}\right)}^{-1}$$,

for r=0,…, m; where **A***^f^* = **I** − **A***^f^* and I is the identity matrix.

Therefore,

(11) $${\bar{A}}_{ij}^{f}=\{\begin{array}{c} 1-\sum_{r=1}^{m}A_{ij}^{r}e^{-J2\pi fr},if(i=j)\\ -\sum_{r=1}^{m}A_{ij}^{r}e^{-J2\pi fr},otherwise \end{array}$$,

where $A_{ij}^{r}$ denotes the coefficients that describes the relationship between the present of time series **w_*i*_** and the past of **w_*j*_** .

DTF represents the causal influence from channel j to channel i which is defined by Equation (12) .

(12) $${\left({è}_{j\to i}^{f}\right)}^{2}={\left|h_{ij}^{f}\right|}^{2}$$,

where $h_{ij}^{f}$ is (*i, j*)*^th^* element of H^f^.

The normalized DTF is presented as Equation (13) .

(13) $${\left({\tilde{a}}_{j\to i}^{f}\right)}^{2}=\frac{{\left|h_{ij}^{f}\right|}^{2}}{\sum_{j=1}^{n_{c}}{\left|h_{ij}^{f}\right|}^{2}}$$,

whereby H^f^ is estimated using Equation (10) .

To estimate A, Equation (7) should be multiplied by **W̄***^n^*^−^*^i^* and after taking the expectation, the Yule Walker Equation is obtained as shown in Equation (14) .

(14) $$\sum_{r=1}^{m}A^{r}R^{i-r}=R^{i}$$,

where ***R^i^*** = *E*[**W_*n*_** **W̄***^n^*^−^*^i^*] and 1≤*i*≤*m* **W̄***^n^*^−^*^i^* denotes the transpose of **W***^n^*^−^*^i^*.

Note that *E*[**W***^n^*^−^*^i^* **ξ***^n^*] = 0 because the perturbation of the current time is unrelated to previous values of the process and **A***^r^*_s_ are are kept outside the expectance operator because they are deterministic rather than statistical quantities. Partial correlation estimation using unbiased covariance was applied in this study (Schlögl, 2006).

After the normalized DTF *γ*_*j*→*i*_ ∈ *i*^1×*n_f_*^ is computed for all EEG channels, the feature vector $v_{s}^{t}\in i^{n_{v}\times 1}$ is given by $v_{s}^{t}=\left[\gamma_{1\to 1},\gamma_{2\to 1},\ldots \gamma_{j\to i},\ldots \gamma_{n_{c}\to n_{c}}\right]$, where the total number of extracted features is $n_{v}=n_{f}\times n_{c}^{2}$

### 2.3. Generalized Partial directed coherence function

In multichannel EEG analysis, sometimes negative causality occurs at certain frequencies that has no physical interpretation. This problem can be overcome by using Partial Directed Coherence (PDC) function. The PDC describes the direction of information flow between multivariate data in frequency domain. The PDC is based on the decomposition of multivariate partial coherences computed using multivariate autoregressive models. It closely reflects Granger causality by paralleling the definition of Granger causality test estimators. This method allows factoring the classical coherence function (the frequency domain counterpart of correlation analysis) of a pair of structures into two directed coherences; one representing the feed-forward aspect and the other representing the feedback aspect of the interaction (Baccald & de Medicina, 2007). In calculating causality between pairs of electrodes, a negative connection at a certain frequency may not convey any physical information. Therefore, Partial Directed Coherence (PDC) was used to avoid this by modeling of connectivity using a multivariate autoregressive model (Baccald & de Medicina, 2007).

The partial directed coherence from series j to series i, at frequency f can be defined using Equation (15) .

(15) $$\left|\pi_{j\to i}^{f}\right|=\frac{\left|A_{ij}^{f}\right|}{\sqrt{\sum_{i=1}^{n_{c}}{\left|A_{ij}^{f}\right|}^{2}}}$$,

where $A_{ij}^{f}$ has been defined in Equation (11) . Also, *π_j_*_→_*_i_* **∈***i^n_f_×1^* takes values in the interval [0–1].

The main difference between DTF and PDC is related to normalization part as shown in Equations (13) and (15) . DTF is normalized with respect to the structure that receives the signal whereas PDC is normalized with respect to the structure that sends the signal.

After the normalized PDC $\pi_{j\to i}^{f}$ is computed for all the EEG channels, the feature vector $v_{s}^{t}\in^{n_{v}\times 1}$ is given by $v_{s}^{t}=\left[\pi_{1\to 1},\pi_{2\to 1},\ldots \pi_{j\to i},\ldots \pi_{n_{c}\to n_{c}}\right]$, where the total number of extracted features is $n_{v}=n_{f}\times n_{c}^{2}$.

### 2.4. Phase slope index

The PSI estimates the direction of information flow between the multivariate data in the frequency domain. The time delay of τ is considered for information to be propagated from the i^th^ channel to the j^th^ channel. The phase of cross spectrum between the i^th^ channel and the j^th^ channel is a factor of frequency and time delay (2πfτ). The PSI is the derivative of this phase with respect to f. In comparison to Granger causality, the PSI method is insensitive to instantaneous mixtures of independent sources. Furthermore, it gives meaningful results even if the phase spectrum is not linear since it has proper weights contributions from different frequencies (Nolte et al., 2008).

The phase slope index presents directional connectivity between pairs of electrodes in the frequency domain. The propagation time of information flow between channel i and j is denoted as τ. The time delay of τ could be estimated from phase of cross spectrum between channel i and j. Derivative of this phase with respect to f is called PSI, which is not sensitive to transient mixture of sources more stable than Granger causality method (Nolte et al., 2008).

PSI is estimated by applying the cross spectrum of w_i_ and w_j_. However, in an alternative approach, the whole data set, for instance w_i_ is divided into k_1_ segments of duration L_T_ as shown in Equation (16) .

(16) $$w_{i,k_{1}}=\left[w_{i}^{1+(k_{1}-1)L_{T}},\ldots ,w_{i}^{n_{k}},\ldots ,w_{i}^{k_{1}L_{T}}\right]$$,

where $w_{i}^{n_{k}}$ denotes the nk^th^ sample of w_i_.

Then cross spectral density of Hanning-windowed **w***_i, k_*__1__ and **w***_j, k_*__1__ is esimated for each frequency of f as defned in Equation (17) .

(17) $$s_{ij}^{\prime f}=\frac{1}{k_{1}}\sum_{k_{1}}^{k_{2}}z_{i,l}^{f}z^{*}{}_{j,l}^{f}$$,

where $z_{i,l}^{f}$ denotes the Fourier transform of the Hanning windowed **w***_i, k_*__1__ using Equation (4) .

After the cross spectral density is estimated, the complex coherency $c_{ik}^{\prime }\in R^{n_{f3}\times 1}$ is computed using Equation (18) .

(18) $$c_{ij}^{\prime f}=\frac{S_{ij}^{\prime f}}{\sqrt{S_{ii}^{\prime f}S_{jj}^{\prime f}}}$$

Then PSI, **Ø***_ij_* is computed using Equation (19) .

(19) $$\emptyset_{ij}=im\left(\sum_{f\in F_{p}}c_{ij}^{\prime *f}c_{ij}^{\prime f+\delta f}\right)$$,

where im denotes taking the imaginary part, *äf* is the frequency resolution and Fp is the set of frequencies over which the slope is summed. Typically, Fp contains all frequencies but restricted to a specified band in this study.

In order to see how ɸ*_ij_* corresponds to a meaningful estimate of the average slope it is convenient to rewrite Equation (19) as Equation (20) .

(20) $$\Psi_{ij}=\sum_{f\in F}\alpha_{ij}^{f}\alpha_{ij}^{f+\delta f}\sin\left(\Phi \left(f+\delta f\right)-\Phi (f)\right)$$,

where $c_{ik}^{\prime f}=\alpha_{ik}^{f}e^{J\Phi (f)}$, and ${\overset{´}{a}}_{ik}^{f}=\left|c_{ik}^{\prime f}\right|$ are frequency dependent weights.

For smooth phase spectra

(21) sin(Φ(f+δf)−Φ(f))≈Φ(f+δf)−Φ(f)

Therefore, **Ψ***_ij_* corresponds to a weighted average of the slope.

Finally, as for the Granger causality, it is convenient to normalize **Ψ***_ij_* by an estimate of its standard deviation that is done using Equation (22) .

(22) $${\tilde{\Psi }}_{ij}^{\tilde{a}}=\frac{\Psi_{ij}}{std(\Psi_{ij})}.$$

The value of std(**Ψ***_ij_*) is estimated using the Jackknife method and is validated in simulations.

In this study, *Fp* denotes the EEG frequency bands. A filter bank of a number of 4 Hz bands was implemented.

**Figure 4.**
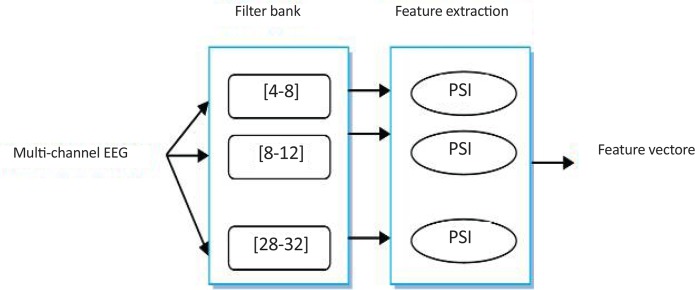
Block diagram of applied filter bank

As shown in Figure 4, the frequency band of [4–32] Hz was divided to 7 parts as follows:

[4–8]|[8–12]|[12–16]|[16–20]|[20–24]|[24–28]|[28–32] Hz.

PSI is computed for each part, as example in part b

Ψ˜ija˜=[0Y˜ija˜Y˜bjia˜0],

where *_b_Y*^ã^*_ij_*=−*_b_Y*^ã^*_ij_* . Also, *_b_Y*^ã^*_ij_* denotes the information flow from channel i to channel j in frequency band of part b.

In addition, *_all_Y*^ã^*_ij_=*[*_1_Y* ^ã^*_ij_,...,_b_Y*^ã^*_ij_,...,_7_Y*^ã^*_ij_*] denotes the PSI for all frequency bands and *_all_Y*^ã^*_ij_***∈***i^7^*^×^*^1^* is ex tracted for all channels. The feature vector extracted is given by $v_{s}^{t}\in i^{n_{v}\times 1}$, where $n_{v}=7\times n_{c}^{2}$ and vst=[Y˜all11a˜,…,Y˜allija˜,…,Y˜allnccnca˜].

After the feature vectors were extracted, some of the extracted features may be irrelevant or redundant and may have a negative effect on the accuracy of the classifiers. Therefore, a number of significant features should be selected. Feature ranking was performed using the class separability criteria (Guyon & Elisseeff, 2003; Reyes-Aldasoro & Bhalerao, 2006). Afterward, the most significant features were labeled and classified.

**Figure 5.**
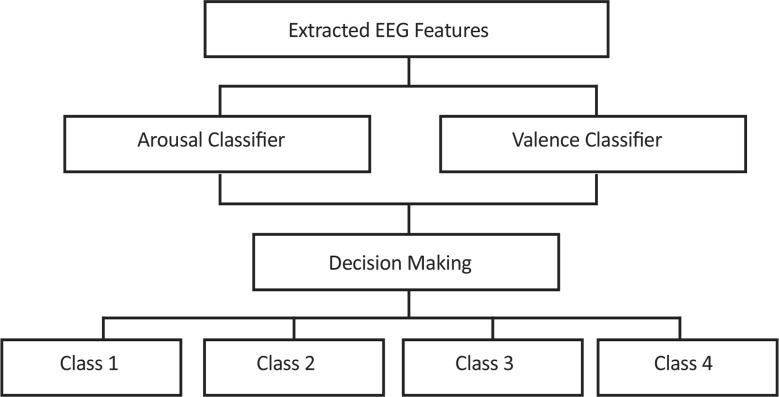
Structure of classifier

**Figure 6.**
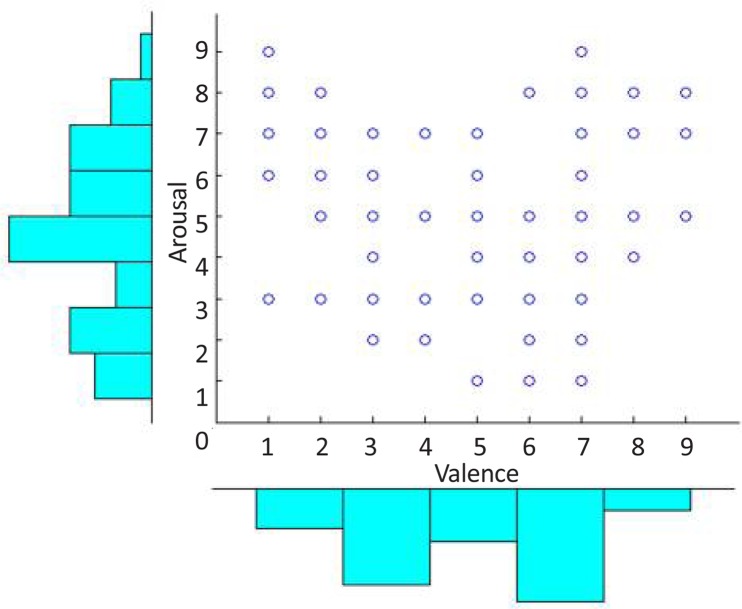
Subjects’ SAM distribution

According to Figure 5, the valence and arousal levels are classified separately. The boundaries between different classes are determined from the subjects’ answers to the SAM questionnaire. The SAM answers are 2 dimensional labels that one dimension denotes the valence level and another one the arousal level. The SAM answers have 1 level of uncertainty, for instance *Valence* =5±1 denotes the neutral level (Valence=5). According to the SAM answers shown in Figure 6, emotion categories are formed using Valence≤3 (negative) or Valence≥7(positive), and Arousal≤3 (calm) or Arousal*≥*7 (excited). Therefore, binary labeling is done for valence and arousal levels detection. For example, the subjects with Valence*≤*3 or Valence*≥*7 are categorized as valence groups where subjects with Valence*≤*3 are considered as class 1 and subjects with Valence*≥*7 are labeled as class 2. The similar process was performed for labeling of arousal classes as well. Therefore the binary classes were configured as given by Equation (23) .

(23) $$\tilde{a}\in i^{2\times (n_{e}n_{s})}\Rightarrow \{\begin{array}{l} c_{v}\in i^{1\times n_{s_{v}}}\\ c_{a}\in i^{1\times n_{s_{a}}} \end{array}$$,

where *c_v_* denotes the valence groups labels and *n_s_v__* is the total number of subjects in this category. Similarly, c_a_ denotes the arousal groups labels and *n_s_a__* is the number of subjects in this category.

The extracted features, **ã**, were labeled using **c***_v_* for valence detection as **ã*v*** ∈ **i^n_v_^** **×** **nsv** and using **c**_a_ for arousal detection as **ã**_*A*_ ∈ *i^n_v_^* × *^n_s_a__^*. The labeled input for valence classifier **I**_*V*_ ∈ *i^n_out_^* × *^n_s_v__^* and arousal classifier are **I**_*V*_ ∈ *i^n_out_^* × *^n_s_a__^* defined by Equation (24) .

(24) $$\{\begin{array}{l} I_{V}=f_{r}\left(\gamma_{V}^{t},n_{out}\right)\\ I_{A}=f_{r}\left(\gamma_{A}^{t},n_{\mathrm{out}}\right) \end{array}$$,

where t denotes the transpose of matrix, and *n_out_* = 24 is the number of output features.

### 2.5. Classification

A wide range of classifiers can be applied to derive the affective states. Since four classes of emotions are studied, various multiclass classification schemes can be used such as all-together, one-against-one, one versus rest, and hierarchical model. In this study, the Figure 5 model was applied.

The model is constructed using two binary classifiers of the same type: one classifier is trained to classify valence and the other is trained to classify arousal. In this model, the four-class classification is decomposed into two-level nested binary classifiers based on arousal-specific level and valence-specific level. Two types of classifiers are applied using the applied model on the extracted features: 1. K-Nearest Neighbor (KNN) and 2. Support Vector Machine (SVM). The study classifier scheme is based on two similar binary classifiers, one for discrimination of valance level (positive, negative) and the other one for discrimination of arousal level (calm, excited). The classification accuracy of this scheme was calculated using either K-Nearest Neighbor classifier (KNN) or the Support Vector Machine (SVM). The KNN classifier is a supervised learning algorithm to categorize objects based on the closest training examples in the feature space (Filipek et al., 1999). In contrast, the SVM classifier finds a separating hyperplane with the maximal margin between two classes of data (Shawe-Taylor & Cristianini, 2000). In this study, the features were mapped using Gaussian radial basis function into the kernel space with the sigma of the RBF kernels set to 3.5 for arousal and 6 for valence level detection.

## 3. Results

**Figure 7.**
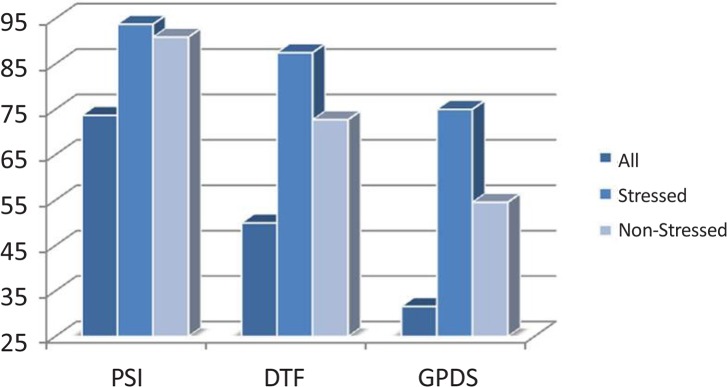
Classification accuracy using SVM classifier

**Figure 8.**
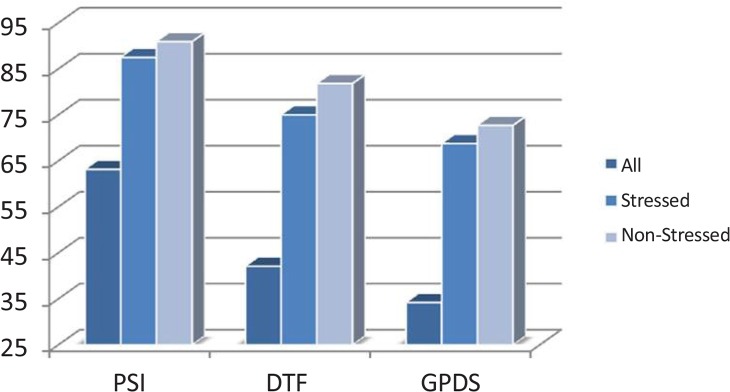
Classification accuracy using KNN classifier

Four classes of emotions including negative and positive emotions from valence dimension and calm and highly exited emotions from arousal dimension were investigated. The classification accuracies using three different features and two classifiers are shown in Figure 7 and Figure 8. The results of KNN and SVM classifiers are based on leave-one-out method. Programming was done using MATLAB and “BioSig” toolbox to extract the connectivity features (Schlögl & Brunner, 2008).

**Figure 9.**
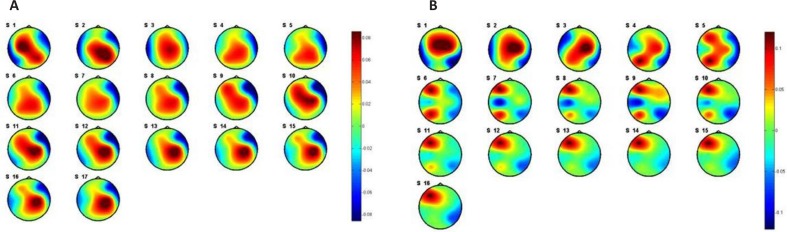
Pattern of averaged PSI for stressed (a) and stress-free (b) subjects in positive emotional states (Valence≥7)

**Figure 10.**
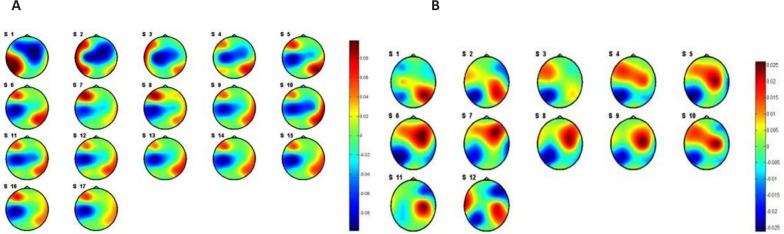
Pattern of averaged PSI for stressed (a) and stress-free (b) subjects in negative emotional sates (Valence≤3)

Among the three implied methods, PSI feature yields better classification accuracy. The results show a significant improvement in the classification accuracy (*μ* =68.42) by removing the effect of the internal factor through categorizing the subjects to stressed (*μ_s_* = 90.62) and stress-free (*μ_n_* = 90.90) groups. These results indicate that there are patterns of brain regions connectivity (wiring structures) during the perception of external stimuli that chronic stress can change them. To clarify the concept of connectivity between brain regions, the flux average of each EEG channel was evaluated using PSI. Sum of PSI of 2 s EEG signal in duration of 50 s was computed for each channel. The patterns of the average PSI for all channels in positive and negative emotiTable 1. P-values of t test between stressed subjects vs. stress-free ones in positive and negative emotional stateshown in Figure 9 and Figure 10, respectively.

Statistical analysis of the average PSI results using t test shows significant changes on C3, C4, F4, P4, T8 for positive emotions and C3, F4, P3, P4, T8 for negative emotions. A 2-tailed unpaired t test considering unequal variance for the two groups was computed. Table 1 shows the acquired P values from t test. The P values show changes in average PSI on C4 dominates during positive emotions while changes in channel P3 dominates in negative emotions.

**Table 1.** P-values of t test between stressed subjects vs. stress-free ones in positive and negative emotional states

**Valence**	**C3**	**C4**	**F4**	**P3**	**P4**	**T8**
≥7	1.1e–13	0.03	1.3e–5	0.67	0.0006	0.002
≤3	5.8e–15	0.67	9.6e–5	0.003	0.04	0.0003

**Figure 11.**
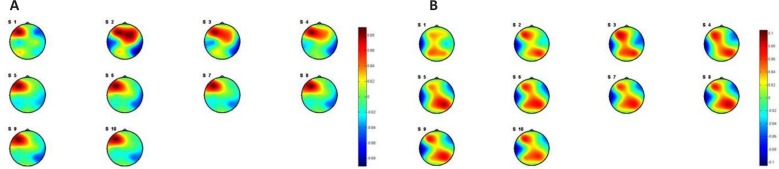
Pattern of averaged PSI for stressed (a) and stress-free (b) subjects in resting state

Regarding the present Table 1. P-values of t test between stressed subjects vs. stress-free ones in positive and negative emotional statesata in Figure 9 and Figure 10, there is no consideration on the arousal levels and its effect on the valence levels. The same comparison was done for the resting state (eyes close condition). Figure 11 shows the results of the averaged PSI for 5 minutes of EEG signals. Table 2 shows the P value of effective channels extracted using t test on unpaired data.

**Table 2.** P values of t test between stressed subjects vs. stress-free ones in resting state

	**C3**	**P3**	**P4**	**T7**
P	1.5e–6	1.6e–5	0.0001	0.015

**Figure 12.**
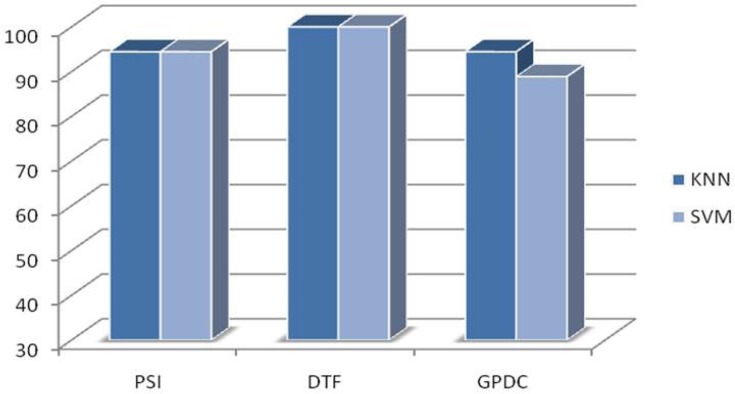
Classification accuracy using SVM and KNN classifiers

The results shown in Figure 11 indicate the possibility of using the connectivity features of EEG in the resting state for detection of chronic stress. To investigate such an idea, the subjects were classified to stressed and stress-free group based on these features. The classification accuracy of the system for stress level detection applying the SVM and the KNN is shown in Figure 12.

The results shown in Figures 7, 8, and 12 emphasize that either in resting state or emotional states the patterns of connectivity among the brain regions are changed in response to stress level. Note that this study was limited to adults aged from 18 to 30 years old, so such results cannot be generalized to a younger or older population. In addition, the EEG signals below 4 Hz or above 32 Hz were not evaluated. The results are based on 8 brain regions that precision of results may be tolerated by applying more or less number of EEG electrodes.

## 4. Discussion

Chronic stress causes a series of chemical compounds releases and reactions in our bodies. Therefore, stress as an internal stimulus can potentially influence the responses to external stimulus. In this study changes in brain activity in response to external emotional stimuli considering the subject’s stress level was investigated. The effective connectivity between eight brain regions based on the EEG signals were correlated to four emotional states. The classification accuracy of the emotion recognition system improves by separating the subjects to two different levels of stress. The effect of stress on connectivity pattern of brain regions was also tested in resting state. The results show that stress can change connectivity patterns among different brain regions either in resting state or emotional stimuli perception. However, impact of stress on changing the pattern of connectivity is subject-dependent.
